# CircRNA-mediated regulation of cardiovascular disease

**DOI:** 10.3389/fcvm.2024.1411621

**Published:** 2024-11-26

**Authors:** Ke-yun Cheng, Si-wei Wang, Tian Lan, Zhu-jun Mao, You-yao Xu, Qing Shen, Xi-xi Zeng

**Affiliations:** ^1^ Panvascular Diseases Research Center, The Quzhou Affiliated Hospital of Wenzhou Medical University, Quzhou People's Hospital, Quzhou, China; ^2^ College of Pharmaceutical Sciences, Zhejiang Chinese Medical University, Hangzhou, China; ^3^ Department of Cardiovascular Surgery, The Quzhou Affiliated Hospital of Wenzhou Medical University, Quzhou People's Hospital, Quzhou, China

**Keywords:** cardiovascular diseases, circRNA, risk, cerebrovascular disease, diagnostic

## Abstract

Cardiovascular diseases (CVDs) encompass a range of disorders affecting the heart and blood vessels, such as coronary heart disease, cerebrovascular disease (e.g., stroke), peripheral arterial disease, congenital heart anomalies, deep vein thrombosis, and pulmonary embolism. CVDs are often referred to as the leading cause of mortality worldwide. Recent advancements in deep sequencing have unveiled a plethora of noncoding RNA transcripts, including circular RNAs (circRNAs), which play pivotal roles in the regulation of CVDs. A decade of research has differentiated various circRNAs by their vasculoprotective or deleterious functions, revealing potential therapeutic targets. This review provides an overview of circRNAs and a comprehensive examination of CVDs, the regulatory circRNAs within the vasculature, and the burgeoning research domain dedicated to these noncoding RNAs.

## 1 Introduction

Cardiovascular disease (CVD) remains a predominant cause of mortality globally, imposing significant economic strain on healthcare systems. The “China Cardiovascular Disease Health and Illness Report 2021” delineates a staggering figure of 330 million individuals grappling with cardiovascular ailments in China. Concurrently, data extracted from NHANES (2017 to March 2020) indicates a prevalence rate of cardiovascular diseases (encompassing coronary heart disease, heart failure, stroke, and hypertension) at 48.6% among adults aged 20 and above in the United States, which translates to an estimated 127.9 million affected individuals in 2020, with a noted increment correlating with advancing age across both genders (1). Extensive scientific and clinical research efforts are directed towards improving the diagnosis, treatment, and prognosis of CVD. Despite these efforts, there remains a substantial gap in the availability of effective therapeutic interventions.

Chronic diseases such as diabetes and hypertension often share etiological risk factors, laying a common pathogenic groundwork that is closely linked with CVDs, including endocrine abnormalities. Advancements in RNA sequencing and bioinformatics have highlighted circRNAs as pivotal players in biological processes like carcinogenesis, angiogenesis, and immune responses, positioning them as promising biomarkers and therapeutic targets (2). Recent research has extended circRNA studies to CVDs, revealing their diverse roles: circSCMH1 has been implicated in enhancing vascular repair through FTO-mediated m6A methylation of Plpp3 following stroke (3); circMET silencing has been shown to suppress tip cell specialization and retinal angiogenesis (4), and hsa_circ_0076631 has been reported to modulate caspase-1-induced pyroptosis via miR-214-3p targeting in diabetic cardiomyopathy (DCM) (5). Furthermore, the knockdown of circRNA circ_0071269 has been demonstrated to confer protection against DCM through the microRNA-145/gasdermin A axis.

In our comprehensive review, we provide an overview of circRNAs and delineate the spectrum of CVDs, with a focus on the modulatory impact of circRNAs on these conditions. We delve into the molecular intricacies of circRNA-mediated regulation in vascular pathology and critically evaluate emerging insights into their diagnostic and therapeutic potential. Our synthesis aims to deepen the understanding of CVDs and propose innovative therapeutic avenues to mitigate the global burden of blindness.

## 2 Overview of CircRNAs

### 2.1 CircRNA biogenesis

In eukaryotes, most circRNAs are formed through back-splicing (6, 7). This process is facilitated by inverted Alu repeats or complementary sequences in the introns flanking the circularized exon, which bring the downstream 5′ splice site close to the upstream 3′ splice site, thereby promoting intron pairing and back-splicing (8, 9). A single gene can generate multiple circRNAs via selective back-splicing at various 5′ and 3′ splice sites (10). In a study of mouse heart tissue, 1,283 unique circRNAs were discovered across six samples (11). Roughly half of these host genes produced only one type of circRNA, while the others generated between two and nine variants. The titin gene was particularly notable, producing 38 distinct circRNAs through complex splicing events in its I-band region (11). CircRNAs are classified by composition: exonic circRNAs (EcircRNAs) contain only exons, while exon–intron circRNAs (EIciRNAs) include both exons and introns. In eukaryotic cells, circRNAs are generated by the interaction of intron pairing, RNA-binding proteins (RBPs), lariat structures, and TSEN-RtcB-mediated circularization. Despite their diversity, all circRNAs share a key feature: they lack a covalently closed circular structure with a 5′ cap and a 3′ poly(A) tail. This absence results in circRNAs having longer half-lives (19–24 h) compared to their linear counterparts (4–7 h) that share the same nucleotide sequence (12).

### 2.2 The molecular functions of circRNAs

Eukaryotic cells express thousands of distinct circRNAs, which are increasingly recognized for their diverse molecular functions, such as acting as miRNA sponges, regulating protein activity, and serving as templates for protein synthesis (13).

CircRNAs can sequester miRNAs by base-pairing with them, thus preventing these miRNAs from binding to their target mRNAs and mediating gene silencing. This “sponge” function allows mRNAs to evade miRNA-induced regulation. For instance, CDR1as (ciRS-7) is abundant in human and mouse brains and acts as a miRNA sponge (14). Similarly, circ-ITCH sequesters miR-7, miR-20a, and miR-214 (15), while circ-DAB1 targets miR-1270 and miR-944 (16). Other examples include circCCDC66, which absorbs miR-33b and miR-93, circ-Foxo3, which binds eight distinct miRNAs, and circHIPK3, which provides binding sites for nine different miRNAs (17). The sponging activity of circRNAs can vary depending on the tissue or cell type. For example, CircSLC8A1 sponges miR-130b and miR-494 in bladder cancer (18) and miR-133a in cardiac hypertrophy (19). CircZNF609 similarly acts on different miRNAs, sponging miR-615-5p in retinal cells (20) and miR-150-5p in megacolon (21).

Beyond miRNA sponging, circRNAs can regulate protein functions by acting as baits, adapters, scaffolds, or sponges (22). Their roles include (i) regulating transcription or splicing via R-loop formation, (ii) maintaining stem cell properties or inhibiting PKR through short hairpin structures, (iii) modulating gene activity by isolating or competing with proteins, (iv) altering bioactivity by forming complexes with proteins, and (v) serving as templates for protein synthesis.

CircRNAs lack the 5′ cap and 3′ poly(A) tail characteristic of most mRNAs, necessitating internal ribosome entry for translation initiation. Recent studies support the internal initiation of translation on circRNAs (23). For instance, the untranslated region (UTR) of circZNF609 acts as an internal ribosome entry site (IRES), functioning in a splicing-dependent manner, differing from traditional IRES elements (24). Additionally, Pamudurti et al. found that circMbl undergoes cap-independent translation, with both its UTR and reverse complement facilitating this process, indicating that RNA structure, not sequence, may drive internal initiation (25). Zhang et al. identified circPINT exon2, a circRNA from a long non-coding RNA, which contains an IRES enabling translation of a short 87 amino acid open reading frame (ORF) (26).

Future research is expected to reveal even broader roles for circRNAs, expanding our understanding of their molecular functions.

## 3 Overview of cardiovascular diseases

### 3.1 Cardiovascular diseases

As the principal cause of death worldwide, CVD imposes a significant health burden, with its prevalence and mortality escalating, especially among those over 40 (27). In 2020, CVD was responsible for approximately 19.05 million deaths globally, an increase of 18.71% since 2010 (1). Interventions targeting vascular risk factors and curtailing the progression of atherosclerosis have been shown to decrease coronary events, thereby diminishing the long-term incidence of cardiovascular mortality and terminal heart disease. Atherosclerosis underpins CVDs and is driving a surge in the incidence, prevalence, and mortality of atherosclerotic cardiovascular disease (ASCVD) within the Chinese demographic (28). Atherosclerosis (AS) is characterized by ischemic lesions resulting from lipid accumulation on the luminal surface of arterial vessels, driven by elevated blood lipid levels. This process leads to morphological alterations of the vessel's inner wall, culminating in luminal narrowing or occlusion. Atherosclerosis progresses through four distinct stages. Stage I (fatty streak stage) is marked by the appearance of yellowish spots or streaks, approximately 1–2 mm wide, on the arterial intima, which may be flat or slightly elevated. Stage II (fibrous plaque stage) involves the continued deposition of lipids, including phospholipids, lipoproteins, and cholesterol, leading to the enlargement and softening of these streaks into plaques. Stage III (atheromatous plaque stage) is characterized by the development of gray-yellow plaques that elevate the endothelial surface, eventually becoming porcelain-white due to increased collagen fiber deposition and vitreous degeneration. Stage IV (complicated lesion stage) involves the rupture of newly formed capillaries within the plaque, resulting in intraplaque hemorrhage and acute luminal obstruction. This stage may also feature surface necrosis, ulceration, calcification, and thrombosis, leading to organ ischemia and dysfunction. Additionally, the weakening of the medial smooth muscle beneath the plaque can result in aneurysm formation, with potential rupture causing hemorrhage.The pathogenesis of AS is multifaceted, encompassing genomic and proteomic factors, homocysteine accumulation, inflammatory responses, oxidative stress, lipid infiltration, and thrombosis.

### 3.2 Cerebrovascular diseases

Cerebrovascular diseases, which disrupt cerebral blood flow, manifest acutely as strokes—sudden events precipitated by vessel rupture or blockage leading to brain damage. Severity ranges from recoverable dysfunction to coma, death, or persistent disability, predominantly afflicting those over 40 (29), though young adults are not exempt, particularly from cerebral embolism and subarachnoid hemorrhage (30, 31). Risk factors such as emotional stress (32), overwork (33), blood pressure variability (34), dehydration (35), and certain medical conditions have been implicated, with hypertension, heart disease, hyperlipidemia, and alcohol intake being primary contributors (36). Hypertension management is pivotal for cerebrovascular disease control. The GBD 2019 study reveals increasing stroke occurrences, yet a decline in age-standardized incidence rates (37). A higher Life's Simple 7 score correlates with diminished dementia risk, and cardiovascular disease burden inversely affects cognitive impairment-free life expectancy across ages (38).

### 3.3 Peripheral vascular disease

Peripheral vascular disease, while less prominent in vascular disease discourse than its cardiovascular and cerebrovascular counterparts, demands equal attention due to its prevalence and shared systemic etiology with coronary artery disease. This commonality extends to pathogenic factors and therapeutic strategies. A comparative analysis of MarketScan and Medicare data indicates a discrepancy in clinical management: patients with peripheral artery disease (PAD) are less frequently prescribed statins and are less likely to achieve LDL-C targets below 70 mg/dl than those with coronary artery disease (39). Additionally, in individuals aged 60 to 74, risk factors such as male sex, hypertension, and family history significantly influence the incidence of thoracic aortic aneurysm (40).

### 3.4 Microangiopathy

Microvessels play a crucial role in maintaining human health, serving as essential conduits for blood transport and facilitating the exchange of materials between blood and tissues. The structural and functional integrity of microvessels is vital for the optimal performance of organs such as the heart, brain, and kidneys. Microvascular damage represents a systemic pathology, underpinning the development of cardiovascular, cerebrovascular, and renal diseases. Protecting microvascular endothelial cells is central to addressing microvascular lesions, forming a cornerstone in the prevention and treatment of major conditions like cardiovascular diseases, cerebrovascular disorders, and diabetes mellitus. The progression of microangiopathy typically involves initial functional changes in microcirculation, followed by endothelial damage (41) and basement membrane thickening (42). These alterations lead to morphological changes in microvessels, including twisting, malformation, and knotting. Additionally, direct damage from bacteria and endotoxins can result in microaneurysm formation. Concurrently, microvascular walls become rough, lumens narrow, elasticity diminishes, and vasodilation occurs. These changes, coupled with metabolic abnormalities such as increased blood viscosity, lead to blood flow stagnation, cell aggregation, and pronounced exudation or hemorrhage, increasing the fragility of microvascular walls.

## 4 Risk factors for cardiovascular diseases

Each year, the heart association aggregates vital statistics concerning heart disease, stroke, and associated cardiovascular risk factors, offering a comprehensive overview of key health behaviors and metrics. This data set includes critical determinants of cardiovascular health such as blood pressure, dietary habits, cholesterol profiles, glucose regulation, and extends to encompass health-related factors like smoking status, physical activity levels, and body weight, underscoring their collective impact on cardiovascular outcomes. Hypertension inflicts vascular damage through a triad of mechanisms: acute hypertensive episodes precipitate endothelial injury, potentially triggering aortic dissection and vessel rupture; sustained hypertension fosters arterial wall deterioration, atherogenesis, plaque instability, occlusion, and thrombotic events; and persistent elevations in blood pressure provoke microvascular changes, including mesangial expansion, arteriolar hyperplasia, and luminal narrowing, thereby exacerbating the hypertensive state. Furether more, In the United States, diabetes was diagnosed in 10.6% of adults from 2017 to 2020, with peripheral arterial disease and heart failure emerging as the most common initial CVD manifestations in this cohort (1). DM serves as an independent predictor for CVD, which is now the leading cause of death there as of 2022 (43). Notably, lipid-lowering and antihypertensive interventions demonstrate greater efficacy in ameliorating macrovascular complications than glycemic management alone. Empirical evidence establishes a direct link between cigarette smoke exposure and heightened CVD risk, with even minimal exposure and secondhand smoke carrying considerable risk (44, 45). And Chronic kidney disease (CKD) affects over 10% of the global adult population and markedly elevates the risk of cardiovascular disease (CVD), the leading cause of mortality in CKD patients. In 2017, CKD accounted for 1.2 million deaths and contributed to 35 million disabilities worldwide, when adjusted for years of life lost (46). Furthermore, perceived discrimination has been implicated in the onset of gestational diabetes among pregnant women, with obesity partially mediating this relationship. In a paradoxical finding, higher self-reported scores on sleep health were linked to increased heart disease risk (47).

## 5 CirRNAs and cardiovascular diseases

Mounting evidence underscores the significance of circRNAs in the pathophysiology of cardiovascular diseases, where they modulate essential cellular processes such as proliferation, survival, and programmed cell death. The multifaceted roles of circRNAs, from influencing angiogenesis to maintaining vascular homeostasis, position them as potential biomarkers and therapeutic targets in cardiovascular diseases management. This review delves into the intricate ways circRNAs contribute to both vascular quiescence and neovascularization, asserting their profound impact on the diagnostic and therapeutic landscape of cardiovascular diseases, as comprehensively catalogued in Table 1.

**Table 1:** Experimental certificated functions of circRNAs in CVDs.

Diseases	CircRNAs	Cells	Expression in disease	Effects on CVDs	Pathway	References
CVD	circHIPK2	Vascular smooth muscle cells	Upregulated	Promote the Angiotensin II (AngII)-induced VSMC phenotype switching	miR-145-5p	(48)
	circ_0000284	Peripheral blood	Upregulated	As a promising biomarker for the early detection of hypertension	/	(49)
CTEPH	circ-myh8	Pulmonary artery smooth muscle cells	Upregulated	Induce the proliferation and cell-cycle progression	circ-myh8-KAT7-HIF1α	(50)
	hsa_circ_005372	Human plasma samples	Upregulated	Might be associated with the occurrence and regulation of CTEPH	/	(51)
	hsa_circ_003416	Children plasma samples	Downregulated	As a biomarker for PAH-CHD diagnosis	/	(52)
	circWDR37	Pulmonary arterial smooth muscle cells (PASMCs)	Upregulated	Attenuate proliferation and cell-cycle progression	hsa-miR-138-5p	(53)
	circSMOC1	Pulmonary artery smooth muscle cells	Downregulated	CircSMOC1 knockdown promoted proliferation and migration	PTBP1 and miR-329-3p	(54)
	circ-Ntrk2	Mouse hypoxic lung tissues	Upregulated	Alleviated pulmonary vascular remodelling	miR-296-5p-TGF-β1/p38 MAPK	(55)
	circ-Sirt1	Pulmonary artery smooth muscle cell	Downregulated	Represse pulmonary artery smooth muscle cell proliferation, migration and autophagy	microRNA-145-5p/protein kinase-B3	(56, 57)
	circNFXL1	Human Pulmonary Artery Smooth Muscle Cells	Downregulated	Effect of hypoxic pulmonary vasoconstriction	miR-29b-2-5p	(58)
CHD	hsa_circ_0001445	Human plasma samples	Upregulated	Potential non-invasive biomarkers	/	(59)
	hsa_circRNA_0000284	Vascular smooth muscle cell	Upregulated	Regulated of vascular smooth muscle cell proliferation	ELAVL1 and miR-939	(60, 61)
CAD	hsa_circ_0089378	Plasma samples	Upregulated		hsa-miR-130a-3p-TRPM3	(62)
	hsa_circ_0126672		Upregulated		miR-145-5p	(63)
	hsa_circ_0030042	Human umbilical vein endothelial cells	Downregulated	Inhibit ox-LDL-induced abnormal autophagy of HUVECs and maintain plaque stability	eIF4A3	(64)
AMI	circABCA3	Ardiac microvascular endothelial cell	Upregulated	Silencing of circABCA3 enhanced proliferation, migration, and angiogenesis and inhibited apoptosis	miR-671-5p-PCSK9	(65)
MF	circ_0002295	Cardiac myofibroblasts	Upregulated	Knockdown of circ_0002295 effectively suppressed the proliferation, migration and MF progression	miR-1287-CXCR2	(66)
DCM	circDICAR	Diabetic cardiomyocyte	Downregulated	Knockdown of DICAR enhanced the diabetic cardiomyocyte pyroptosis	DICAR-VCP-Med12	(67)
	circ CDR1as	Diabetic cardiomyocyte	Upregulated	knockdown of CDR1as could improve the apoptosis	ALKBH5-FOXO3-CDR1as/Hippo signaling pathway	(68)
	CACR	Diabetic cardiomyocyte	Upregulated	Silencing of CACR enhanced pyroptosis in cardiomyocytes	CACR/miR-214-3p/caspase-1	(69)
	CircMAP3K5	Diabetic cardiomyocyte	Upregulated	Exacerbates high glucose-induced cardiomyocyte apoptosis	miR-22-3p-DAPK2	(70)
CIS	Circ DLGAP4	Transient middle cerebral artery occlusion mouse stroke model	Downregulated	Influenc endothelial-mesenchymal transition	miR-143	(71)
	circHECTD1	Astrocyte	Downregulated	Decrease infarct areas, attenuated neuronal deficits, and ameliorated astrocyte activation	miR-142-TIPARP	(72)
	circUCK2	Astrocyte	Upregulated	Amelioration of neuronal injury	miR-125b-5p-GDF11	(73)
	circSHOC2	Astrocyte	Upregulated	Enhance neuronal autophagy and mitigates ischemic brain damage	miR-7670-3p-SIRT1	(74)
	circPRDX3	Neuronal	Downregulated	Boost cell survival and lowering apoptosis	miR-641-NPR3	(75)
ICH	circAFF2	Neuronal	Upregulated	Promote neuronal cell injury	miR-488-ca-3	(76)
RVD	circRNA_010383	Tubular epithelial cells	Downregulated	Promote proteinuria and renal fibrosis	miR-135a	(77)
	mmu_circ_0001295	Adipose-derived mesenchymal stem cells	Upregulated	Improve sepsis outcomes		(78)
OND	circRSU1	Human retinal endothelial cells	Upregulated	Attenuate diabetic-induced retinal vascular anomalies	miR-345-3p-TAZ	(79)
	cGlra2	Retinal ganglion cells	Upregulated	Decrease retinal cell apoptosis, enhanced visual acuity	miR-144-BCL2L11	(80)

### 5.1 Roles of circRNAs in cardiovascular disease

Cardiovascular disease stands as the principal cause of mortality worldwide, with its incidence and fatality rates escalating post-40 years of age. At the cellular level of cardiac and vascular tissues, stem cells undergo proliferation and differentiation to give rise to precursor cells, which subsequently differentiate into the major cell types of the heart and blood vessels, including cardiomyocytes, smooth muscle cells, cardiac fibroblasts, and endothelial cells, thus forming the foundational components of the cardiovascular system. Cardiovascular disease encompasses a spectrum of conditions affecting the function of the heart and blood vessels, with primary types including coronary heart disease (involving the vessels supplying the myocardium and leading to angina pectoris and myocardial infarction/heart failure due to compromised myocardial perfusion, accounting for a significant proportion of cardiovascular disease cases), cerebrovascular disease (involving vessels supplying the brain, encompassing stroke and transient ischemic attacks), peripheral arterial obstructive disease (involving arterial disease in the extremities), rheumatic heart disease (resulting from damage to the heart muscle and valves due to rheumatic fever caused by streptococcus bacteria), congenital heart disease (comprising structural malformations present at birth), deep vein thrombosis and pulmonary embolism (involving the presence of blood clots in the leg veins that can dislodge and migrate to the heart and lungs), and aortic coarctation (encompassing thoracic and abdominal aortic aneurysms). The risk factors for these diseases are closely associated with genetic or environmental factors, in addition to age, lifestyle, and metabolic disorders. Recent insights highlight the pivotal functions of circRNAs and their modulatory mechanisms in the CVD landscape, suggesting novel molecular underpinnings and potential therapeutic targets.

#### 5.1.1 Coronary heart disease

Coronary heart disease, formally known as coronary atherosclerotic heart disease and often referred to as ischemic heart disease, arises from atherosclerosis in the coronary arteries, resulting in myocardial ischemia and hypoxia. The coronary arteries, named for their crown-like shape, are the sole blood vessels supplying blood to the heart. Atherosclerotic changes in these arteries, consistent with systemic vascular hardening, lead to impaired blood circulation to the myocardium, manifesting as myocardial ischemia and hypoxia, which are hallmark features of coronary heart disease. CircRNAs have gained prominence as potential non-invasive biomarkers for CHD diagnosis and prognosis. Recent studies have also explored hsa_circ_0001445 levels in peripheral blood leukocytes (PBLs) of CHD patients, examining its association with clinical features (81). Additionally, differential expression of hsa_circRNA_0000284 in PBLs from CHD patients compared to healthy individuals suggests its involvement in the regulation of vascular smooth muscle cell proliferation, a key aspect of CHD pathogenesis (60, 61). Concurrently, circRNAs have been proposed as predictive biomarkers for the efficacy of Xue-Fu-Zhu-Yu capsules in coronary heart disease (82). The circRNA_0031672/miR-21-5p/PDCD4 axis is implicated in mitigating ischemia/reperfusion injury in myocardial cells (83).

#### 5.1.2 Coronary artery disease

Coronary artery disease (CAD), primarily resulting from atherosclerosis-induced reduction in coronary blood flow, is the leading cause of mortality in both men and women, responsible for approximately one-third of all deaths globally (84). This burden is particularly pronounced in resource-limited regions. The mortality rate from CAD is approximately five times higher in men than in women, although this disparity diminishes with advancing age (85). For instance, circRNAs such as hsa_circ_0089378 and hsa_circ_0126672 have been implicated in the post-transcriptional enhancement of gene expression through miRNA sequestration in CAD contexts (62, 63). Others, including hsa_circ_0000280 and circ-SATB2, are identified as modulators of vascular smooth muscle cell proliferation, a critical process in vascular remodeling (60, 61). Moreover, hsa_circ_0030042 emerges as a stabilizing factor against aberrant autophagy in endothelial cells via the MiR-616-3p/RFX7 Axis, thereby promoting plaque stability (64). Bioinformatics approaches have underscored these circRNA expression alterations in CAD (86, 87), highlighting the necessity for rigorous functional analyses and elucidation of underlying mechanisms (88, 89). Additionally, small extracellular vesicle-derived circRNAs are emerging as gene expression regulators and CAD risk predictors (65). Research into m6A-modified circRNAs has furthered our understanding of their role as potential CAD biomarkers (90, 91). Lastly, circRNA-3302 has been associated with endothelial-to-mesenchymal transition in Kawasaki disease, which may predispose patients to coronary artery aneurysms (92).

#### 5.1.3 Cardiomyopathy

Myocardial fibrosis (MF), a key pathological component of hypertension, myocardial infarction, and heart failure, has been increasingly associated with the regulatory role of circRNAs. Recent research, such as that conducted by Ma et al. (66), underscores the influence of circ_0002295 on MF progression via modulation of the miR-1287/CXCR2 axis. These findings not only enhance our understanding of MF pathogenesis but also highlight the potential of circRNA-targeted strategies for MF management.

Dilated Cardiomyopathy (DCM) is defined by the enlargement of the left, right, or both ventricles, accompanied by myocardial hypertrophy. This condition results in impaired ventricular systolic function, which may occur with or without the presence of congestive heart failure. Recent studies have illuminated the role of circRNAs in DCM, with Dong et al. (93) identifying 58 differentially expressed circRNAs in BKS-db/db knockout mice heart tissues, and Costa et al. (94) finding four highly expressed circRNAs in DCM patient plasma samples. Yuan Q et al. (67) proposed circDICAR and its derivative, DICAR-JP, as potential DCM therapeutics, functioning through the degradation of DICAR-VCP-Med12. Shao et al. (68) underscored the importance of the ALKBH5-FOXO3-circ CDR1as/Hippo pathway and m6A methylation in DCM. Yang F et al. (69) highlighted the therapeutic potential of the CACR/miR-214-3p/caspase-1 pathway, while Shen et al. (70) showed that CircMAP3K5 exacerbates high glucose-induced cardiomyocyte apoptosis via the miR-22-3p/DAPK2 axis. In hypertrophic cardiomyopathy (HCM), Guo et al. (95) identified hsa_circ_0043762, hsa_circ_0036248, and hsa_circ_0071269 as potential key regulators, and Liu T et al. (96) implicated hsa-circRNA-100053-hsa-miR-455-5p-TRPV1 and hsa-circRNA-005843-hsa-miR-188-5p-SPON1 interaction pairs in atrial fibrillation (AF) pathophysiology.

In the quest to elucidate the molecular underpinnings of cardiovascular diseases, several studies have shed light on the significance of circRNAs. Zheng et al. (97) profiled circRNA expression alterations tied to autophagy in a mouse model of acute sepsis, revealing potential regulatory mechanisms. Zhu J et al. (98) uncovered the role of EV-circITGB1 in dendritic cell maturation and subsequent cardiac damage through the miR-342-3p/NFAM1 axis, implicating it in the pathogenesis of AMI. Concurrently, Zhu Y et al. (99) pinpointed a decrease in circSNRK in myocardial infarction-afflicted rats, which modulates cardiomyocyte apoptosis and proliferation by sponging miR-103-3p and upregulating SNRK. Garikipati et al. (100) proposed that elevating circFndc3b levels could enhance cardiac function and remodeling post-MI. Complementing these findings, Lin et al. (101). Sonnenschein et al. (102), and Sun et al. (103) collectively demonstrated the promise of circRNAs as circulating biomarkers across various cardiomyopathies. Collectively, these studies advance our understanding of circRNAs in the context of cardiovascular pathology and highlight their potential as both therapeutic targets and diagnostic tools.

#### 5.1.4 Cardiac hypertrophy

Cardiac hypertrophy, a condition often arising from hypertensive left ventricular hypertrophy and congestive heart failure, is intricately regulated by circRNAs (104, 105). Recent research elucidates a circRNA-centric network modulating myocardial hypertrophy (106). For instance, circRNA_000203 is upregulated following Ang-II infusion, exacerbating cardiac hypertrophy and dysfunction in transgenic mice (107). Concurrently, circRNA_0001006 promotes hypertrophy by inhibiting miR-214-3p, thereby increasing PAK6 (87). whereas heart-related circRNA (HRCR) mitigates hypertrophy by sponging miR-223 (108). Moreover, circCacna1c and circRNA_0001859 have been implicated in pathological hypertrophy through miR-29b-2-5p/NFATc1 and miR-29b-3p/Ctnnb1 pathways, respectively (109, 110). In contrast, circUtrn is essential for beneficial exercise-induced hypertrophy and protection against ischemia/reperfusion (I/R) injury (111), and C-Ddx is involved in the antihypertrophic memory of exercise preconditioning (112). Targeting circSlc8a1 and circ_0001052 has been shown to attenuate hypertrophy (19, 113), and circNfix downregulation correlates with hypertrophy post-transverse aortic constriction surgery (114). These insights into circRNA-mediated pathways offer promising therapeutic targets for cardiac hypertrophy and failure.

### 5.2 Roles of circRNAs in pulmonary hypertension

Pulmonary arterial hypertension (PAH), a significant complication of congenital heart disease, is marked by increased pulmonary vascular resistance, rapid cell proliferation, and vascular remodeling via metabolic reprogramming. Vascular remodeling, a key mechanism in essential hypertension pathogenesis, is notably influenced by circHIPK2 (48). Recent findings from a cohort study of 300 individuals categorized into normotensive, prehypertensive, and hypertensive groups revealed circ_0000284 as a promising biomarker for the early detection of hypertension and for identifying the progression to its intermediate stage (49).

Chronic thromboembolic pulmonary hypertension (CTEPH), a rare disorder characterized by persistent pulmonary arterial obstruction due to fibrotic thrombi, results in increased vascular resistance, pulmonary hypertension, and potential heart failure (115). Miao et al. (116) have identified key molecules, including mRNAs, miRNAs, and circRNAs, implicated in CTEPH, shedding light on its pathogenesis. Abnormal circRNA expression, such as circ-myh8, is implicated in pulmonary hypertension (PH) onset and progression, influencing histone modification during anti-PH treatment and activating the circ-myh8/KAT7/HIF1*α* pathway to counter PH (50). Dysregulated circRNAs, such as hsa_circ_005372 and hsa_circ_003416, may contribute to PAH pathogenesis by altering host gene expression and downstream targets (51, 52). Furthermore, abnormal pulmonary vessel wall remodeling due to excessive proliferation of pulmonary arterial smooth muscle cells (PASMCs) is a key factor in PAH. The circRNA hsa_circWDR37_016 (circWDR37) is implicated as a crucial regulator of hypoxic proliferative disorder in PASMCs, offering a potential novel therapeutic target for PAH (53).

Pulmonary hypertension is characterized by pathological remodeling of the pulmonary vasculature, a process underscored by the proliferation and migration of PASMCs. Recent literature underscores the pivotal role of circRNAs in this remodeling and in the broader pathology of pulmonary and cardiovascular diseases. A spectrum of circRNAs, including circSMOC1 (54), circ-Ntrk2 (55), circ-Sirt1 (56, 57), and circNFXL1 (58), have been implicated in the metabolic reprogramming of pulmonary artery cells, thereby regulating vascular remodeling in PAH. These findings reinforce the significance of circRNAs in the progression of both CTEPH and PAH. A deeper understanding of circRNA regulatory mechanisms may illuminate novel therapeutic avenues for combating PH.

### 5.3 Roles of circRNAs in cerebrovascular diseases

#### 5.3.1 Cerebral ischemic stroke

Ischemic stroke encompasses cerebral tissue necrosis arising from compromised perfusion secondary to stenosis or obstruction within the cerebrovascular system, notably the carotid and vertebral arteries. Cerebral ischemia manifests across a spectrum: transient ischemic attack (TIA), which lacks infarction; reversible ischemic neurological deficit (RIND), with transient dysfunction; stroke in evolution (SIE), marked by progressive symptomatology; and completed stroke (CS), characterized by established infarction. Each subtype reflects a gradation of ischemic insult severity, with implications for both prognosis and therapeutic intervention.

circRNAs have emerged as pivotal regulators in ischemic stroke pathophysiology, with circ.7225, circ.5415, and circ.20623 identified as potential modulators of cerebral ischemia/reperfusion (CI/R) injury, suggesting their utility as therapeutic targets (117). Investigations into rat models of middle cerebral artery occlusion (MCAO) have delineated a circRNA-miRNA interactome, implicating these non-coding RNAs in stroke mechanisms (118, 119). Complementary studies in murine models have characterized circRNA expression post-focal cerebral ischemia (120) in non-ischemic regions contralateral to cortical infarcts, and following CI/R injury (121, 122). At the mechanistic level, circular RNA DLGAP4 has been shown to mitigate ischemic stroke outcomes by modulating miR-143 and influencing endothelial-mesenchymal transition, crucial for blood-brain barrier integrity (71). Similarly, circHECTD1 acts as a miR-142 sponge to regulate TIPARP expression, thereby modulating cerebral ischemia through autophagy pathways (72). The overexpression of circUCK2 has been associated with reduced apoptosis in cerebral ischemia-reperfusion injury through miR-125b-5p/GDF11signaling (73). Moreover, circ_0000831 (123), circHIPK3 (124), circBBS2 (125), Circular RNA TLK1 (126), circular RNA HECTD1 (127) have been implicated in disease progression through miRNA inhibition, underscoring their potential as molecular targets for intervention.

Recent studies have elucidated the role of circRNAs in neuroprotective mechanisms against ischemic brain injury. Chen W et al. (74) demonstrated that exosome-mediated transfer of circSHOC2 from ischemic postconditioning astrocytes (IPASs) enhances neuronal autophagy and mitigates ischemic brain damage through the miR-7670-3p/SIRT1 axis. Complementing this finding, Chen W et al. (75). identified circular RNA circPRDX3 as a critical player in neuronal fate, modulating survival and apoptosis during ischemic stroke by interacting with miR-641 and NPR3. Additionally, Ren et al. (128) reported an upregulation of Circ-Memo1 in the context of kidney ischemia-reperfusion injury and cerebral hypoxia/reoxygenation (H/R) stress, indicating a potential shared pathway in ischemic pathologies across different tissues. These studies collectively highlight circRNAs as promising molecular targets for therapeutic strategies in ischemic conditions.

#### 5.3.2 Intracerebral hemorrhage (ICH)

Recent advances in noncoding RNA research have highlighted circRNAs as pivotal modulators of transcriptome dynamics in the central nervous system. Liu Z et al. (129) demonstrated through comprehensive cerebral circRNA profiling in intracerebral hemorrhage (ICH) rat models that circRNA expression signatures show higher sensitivity to disease progression compared to mRNAs or miRNAs. This finding was further supported by Kim et al. (130), who explored circRNA expression patterns in a rat ICH model, providing insights into potential therapeutic strategies for ICH. Validation of microarray results for three circRNAs by quantitative real-time PCR (qRT-PCR) was reported by Dou et al. (131), bolstering the reliability of these novel biomarkers. In particular, hsa_circ_0005505, circERBB2, and circCHST12 have been proposed as potential diagnostic biomarkers for ICH (132). Furthermore, Qi et al. (76) elucidated the mechanistic role of circAFF2 in promoting neuronal cell injury in ICH by regulating the miR-488/ca-3 axis. Collectively, these findings underscore the potential of circRNAs as therapeutic agents for mitigating ICH-induced injuries.

### 5.4 Roles of circRNAs in renal vascular disease

Renal vascular diseases encompass a spectrum of pathologies arising from vasculitis, metabolic disorders, thrombosis, and embolism, commonly affecting glomerular capillary collaterals and manifesting as urinary alterations, hypertension, and renal dysfunction. Etiologies include atherosclerosis of renal arteries, fibromuscular dysplasia, aortitis, hypercoagulability from contraceptive use, venous stasis from renal vein compression, and endothelial damage. Embolic sources such as thrombi, cholesterol crystals, and septic emboli further contribute to disease. Histologically, these conditions are characterized by ischemic glomerular changes, tubular atrophy, interstitial single-cell infiltration, fibrosis, capillary stasis, interstitial edema, proliferative fibrosis in vascular walls, and the presence of cholesterol crystal voids.

Diabetic nephropathy (DN), a major vascular complication of diabetes mellitus, significantly contributes to mortality among diabetic patients. A key study by Peng et al. (77) demonstrated the potential therapeutic role of circRNA_010383, which, when overexpressed, inhibited high-glucose-induced extracellular matrix (ECM) accumulation and elevated TRPC1 levels *in vitro*. Notably, kidney-targeted overexpression of circRNA_010383 mitigated proteinuria and renal fibrosis in db/db mice. Furthermore, microvascular dysfunction, a major contributor to mortality in septic patients with multi-organ failure, was shown to be ameliorated by exogenous administration of exosomes from adipose-derived mesenchymal stem cells (ADSCs). The effect was mediated via mmu_circ_0001295 delivery, potentially offering a novel therapeutic avenue for sepsis (78). Renal denervation (RDN), a promising therapy for resistant hypertension (RH), may operate via the upregulation of hsa_circRNA_000367 or downregulation of hsa_circRNA_405119, thereby influencing multiple cellular and molecular pathways (133). Collectively, these findings underscore the potential of circRNAs as therapeutic targets for renal vascular diseases.

### 5.5 Roles of circRNAs in ocular neovascular disease (OND)

Ocular neovascular diseases (ONDs), typified by the pathological proliferation of immature blood vessels, are a predominant cause of vision loss and blindness. Effective diagnostic and therapeutic strategies are critical for managing these conditions. Neovascularization encompasses vasculogenesis and angiogenesis, with the latter divisible into five sequential phases: vascular endothelial cell activation (134, 135), basement membrane and extracellular matrix degradation (136), tip cell migration (137), tubulogenesis (138), and vessel maturation (139). In the context of diabetic retinopathy, circRNA microarray analyses have revealed an upregulation of circRSU1. Lentiviral-mediated knockdown of circRSU1 in human retinal endothelial cells attenuates diabetic-induced retinal vascular anomalies by reducing vascular endothelial growth factor expression, inflammation, and oxidative stress, positioning circRSU1 as a promising therapeutic target (79). Differential expression of 529 circRNAs between diabetic and non-diabetic retinas has been identified, with gene ontology enrichment analysis highlighting associations with ATP binding, extracellular exosomes, and intracellular signaling (140). In glaucoma research, quantification of circular RNA-glycine receptor α2 subunit gene (cGlra2) in the aqueous humor discriminates between glaucoma and cataract patients. cGlra2 silencing confers protection to retinal ganglion cells against oxidative or hydrostatic pressure-induced damage *in vitro* (80). Despite numerous identified therapeutic targets and pathways, further research is imperative to pinpoint the primary target for clinical translation.

## 6 Current diagnostic and therapeutic approaches to cardiovascular diseases

The early detection of high-risk vulnerable plaques is crucial for effective treatment and prognosis in cardiovascular disease. A suite of invasive imaging modalities, including Optical Coherence Tomography, Intravascular Ultrasound, Near-Infrared Spectroscopy, and Fluorescence Lifetime Imaging, are employed to discern plaque characteristics and constituents, whereas non-invasive techniques such as ultrasound, CT, MR, and PET offer alternative evaluation methods. CT imaging provides comprehensive insights into both luminal stenosis and plaque composition, while MR imaging similarly elucidates luminal and plaque characteristics. The integration of OCT and IVUS capitalizes on the strengths of both penetration and resolution, facilitating not only the examination of plaque composition but also the assessment of plaque stability via inflammation markers. The FLIm-IVUS hybrid catheter aids in identifying vascular plaque components, including collagen fibers, calcifications, and lipids, with IVUS integration enabling spatial mapping of these elements. Intravascular Photoacoustic Imaging leverages lipid absorption of pulsed light to generate acoustic signals for plaque characterization, in conjunction with IVUS. Moreover, Indocyanine green specifically targets areas with compromised endothelial barriers, highlighting macrophage activity, lipid accumulation, and intraplaque hemorrhage. Lastly, fiber scanning technologies utilize blue, green, and red laser light to distinguish between different plaque constituents, enhancing the precision of cardiovascular diagnostics.

Arterial inflammation is prevalent among middle-aged adults with subclinical atherosclerosis, and its assessment is pivotal for evaluating patient prognosis. Positron Emission Tomography combined with computed tomography or magnetic resonance, using the tracer 18F-fluorodeoxyglucose, serves as a non-invasive approach to gauge vascular inflammation. The perivascular fat attenuation index offers insights into vascular inflammatory states by quantifying the hydrophilic/lipophilic balance of adipocytes, indirectly reflecting adipocyte size. Magnetic resonance molecular imaging with myeloperoxidase-gadolinium exploits the enzymatic activity of MPO to catalyze oligomerization reactions, enabling the covalent binding of Gd to proteins with phenolic groups, thereby imaging vascular inflammation. Additionally, Surface-Enhanced Raman Spectroscopy imaging detects inflammatory responses in arterial plaques through the intravenous administration of nanoparticles targeting inflammatory molecules, which are tagged with Raman spectroscopy-detectable fluorescent substances. Trophoblastic vessels can now be evaluated using Optical Coherence Tomography, providing novel insights into vascular disease. The risk of CVDs is appraised through mechanomics, focusing on adverse hemodynamic characteristics such as low fractional flow reserve computed tomography and high *Δ*FFRCT, among others. Adverse plaque characteristics, including low attenuation plaque, positive remodeling, and punctate calcification, are also crucial in risk assessment.

The current investigation by Sonnenschein et al. (102) evaluates the expression profiles of circulating circRNAs in HCM patients, positing them as potential biomarkers. Exosomes, pivotal in cerebral ischemia, carry nucleic acids like miRNA, circRNA, and lncRNA that facilitate intercellular communication and contribute to neuroprotection by regulating neural development, angiogenesis, and neuroinflammation inhibition. These vesicles also enhance stroke recovery through mechanisms that bolster neural communication, neuronal and myelin synapse development, neurovascular unit remodeling, and nervous system homeostasis. Moreover, exosomes serve as promising vehicles for targeted delivery of therapeutic agents to lesion sites, owing to their capacity for modification and bioactive substance carriage.

In a recent transcriptomic study, Li S et al. (141) employed RNA sequencing to delineate the circRNA expression landscape in a cohort comprising five patients with large artery atherosclerosis-associated stroke and four non-stroke controls. They discovered that the levels of hsa_circRNA_0001599 were significantly associated with the severity of stroke, as measured by the National Institutes of Health Stroke Scale, and with the volume of cerebral infarction. Receiver operating characteristic analysis of hsa_circRNA_0001599 demonstrated its potential as a diagnostic marker for LAA-stroke, with an area under the curve of 0.805, indicating high diagnostic accuracy (95% CI: 0.748–0.862, *p* < 0.001), and achieving a sensitivity of 64.41% and a specificity of 89.93%. These findings suggest hsa_circRNA_0001599 as a promising circRNA biomarker for LAA-stroke.

## 7 Conclusions and future perspectives

This review comprehensively assesses the advancements in research and therapeutic applications of circRNAs in CVDs, acknowledging their involvement in a myriad of cellular processes and associations with diverse human diseases. Recent innovations in technologies and methodologies have bolstered circRNA identification, functional analysis, and therapeutic exploitation. Yet, in the cardiovascular realm, the intricate regulatory mechanisms governed by circRNAs warrant further investigation. While the majority of research has centered on their role as microRNA sponges, alternative functions and pathways remain underexplored. The burgeoning field of RNA biology and related technological progress have propelled RNA-based therapeutics into a new epoch of diversified development, with circRNAs, owing to their unique structural properties, emerging as promising agents in drug and vaccine innovation. This is particularly evident in the design of circRNA-based drugs for CVD therapy, which stands as a compelling aspect of circRNA research, heralding novel avenues for CVD diagnosis and treatment. Crucially, the potential of circRNA-based vaccines for the treatment and prevention of CVDs remains a largely untapped area of exploration. The unique properties of circRNAs offer a promising foundation for the development of novel therapeutic and prophylactic strategies that could revolutionize CVD management. To actualize this potential, critical challenges such as refining circRNA synthesis techniques and enhancing delivery systems must be addressed.

This review aims to distinguish itself from prior analyses by concentrating on the recent advancements and challenges in developing circRNA-based therapies for cardiovascular disease. The progression in RNA biology and related technologies has led to a more diversified evolution of RNA-based drug design and delivery systems, heralding a new era of rapid development in RNA therapeutics. CircRNA, owing to its unique structural properties, exhibits significant potential in the realm of innovative drug development—encompassing the synthesis of circRNAs with endogenous sequences and the engineering of artificial circRNAs—as well as in vaccine development, surpassing conventional RNA therapeutics. While numerous reviews have chronicled the progress of circRNA research within the cardiovascular system, the swift advancements in this field have continually expanded our understanding of circRNAs and their applications in medical research (142, 143). In this review, we provide a comprehensive overview of the techniques employed or potentially applicable for studying circRNA within the cardiovascular system, highlighting their respective advantages and limitations. This information is intended to guide junior investigators in conducting circRNA research in the cardiovascular context and to advance the field's development. We discuss the current state-of-the-art knowledge regarding circRNA, explore therapeutic strategies, and examine the application perspectives and challenges associated with circRNA in CVD.

The expanding comprehension of RNA's diverse roles has catalyzed the development of novel RNA-based therapeutics, positioning RNA therapy as a promising strategy for previously intractable diseases. A particularly compelling area of circRNA research involves the design of circRNA-based drugs for CVD therapy. As our understanding of circRNA's role in CVD and its potential in RNA-mediated therapy grows, new avenues for CVD diagnosis and treatment have emerged. Nonetheless, significant challenges remain. Efforts to efficiently synthesize circRNA have predominantly relied on *in vivo* overexpression strategies, which risk co-producing linear RNA by-products, potentially compromising the quality and purity of circRNA. Addressing this requires direct delivery of synthesized circRNA to target sites, akin to mRNA therapy. Furthermore, engineered circRNA synthesis is often hampered by the introduction of exogenous fragments, resulting in inefficient cyclization and heightened immune responses. Advancing methods for circRNA circRNA production is essential for exploring circRNA functions and developing new circRNA-based technologies. Additionally, the delivery mode of circRNA presents a critical challenge. For instance, nanoparticles, while promising, may inadvertently trigger immune responses or accumulate in unintended tissues, leading to side effects. Thus, further research to mitigate nanoparticle-related side effects and enhance their safety is vital for the clinical application of circRNA therapeutics.

This review delves into the pathological characteristics and pathogenetic mechanisms of multisite vascular lesions, with a particular focus on the role of circRNAs in CVDs. It further investigates the shared mechanistic traits across these diseases, aiming to identify potential biomarkers and prognostic indicators with broad applicability to vascular pathologies.
